# A Spawn Mobile Agent Itinerary Planning Approach for Energy-Efficient Data Gathering in Wireless Sensor Networks

**DOI:** 10.3390/s17061280

**Published:** 2017-06-03

**Authors:** Huthiafa Q. Qadori, Zuriati A. Zulkarnain, Zurina Mohd Hanapi, Shamala Subramaniam

**Affiliations:** Department of Wireless and Communication Technology, Faculty of Computer Science and Information Technolog, University Putra Malaysia, Serdang 43400, Malaysia; zurinamh@upm.edu.my (Z.M.H.); shamala_ks@upm.edu.my (S.S.)

**Keywords:** mobile agent, data gathering, itinerary planning, spawn mobile agent, wireless sensor network

## Abstract

Mobile agent (MA), a part of the mobile computing paradigm, was recently proposed for data gathering in Wireless Sensor Networks (WSNs). The MA-based approach employs two algorithms: Single-agent Itinerary Planning (SIP) and Multi-mobile agent Itinerary Planning (MIP) for energy-efficient data gathering. The MIP was proposed to outperform the weakness of SIP by introducing distributed multi MAs to perform the data gathering task. Despite the advantages of MIP, finding the optimal number of distributed MAs and their itineraries are still regarded as critical issues. The existing MIP algorithms assume that the itinerary of the MA has to start and return back to the sink node. Moreover, each distributed MA has to carry the processing code (data aggregation code) to collect the sensory data and return back to the sink with the accumulated data. However, these assumptions have resulted in an increase in the number of MA’s migration hops, which subsequently leads to an increase in energy and time consumption. In this paper, a spawn multi-mobile agent itinerary planning (SMIP) approach is proposed to mitigate the substantial increase in cost of energy and time used in the data gathering processes. The proposed approach is based on the agent spawning such that the main MA is able to spawn other MAs with different tasks assigned from the main MA. Extensive simulation experiments have been conducted to test the performance of the proposed approach against some selected MIP algorithms. The results show that the proposed SMIP outperforms the counterpart algorithms in terms of energy consumption and task delay (time), and improves the integrated energy-delay performance.

## 1. Introduction

A wireless sensor network (WSN) is a distribution of hundreds or thousands of sensor nodes that can monitor physical or environmental conditions such as temperature, sound, vibration, pressure, motion, or pollutants [1,2]. These sensor nodes are generally self-powered (like batteries) and limited in memory and processing. The main purpose of the sensor nodes is to sense the information in an area of interest and forward the sensed information periodically to the base station (sink node) for gathering and data processing. Therefore, inefficient forwarding or routing of the information from the sensor nodes to the sink will deplete the energy of nodes, and in most cases, if one node runs out, it will directly affect the connection of WSNs. Several energy-efficient routing protocols, such as low energy adaptive clustering hierarchy (LEACH), hybrid energy-efficient distributed clustering approach (HEED), and energy-efficient opportunistic routing (EEOR) have been proposed to minimize energy consumption and increase the network’s lifetime [3].

In the traditional architecture of data gathering (client–server architecture), the sensor nodes are required to send their sensed data to a sink node via multi-hops. Due to the huge amount of data flows, this process leads to data congestion, increased latency, and high energy consumption—especially for the nodes located near to the sink. This issue has a significant impact on the performance and lifetime of the network.

**Figure 1:**
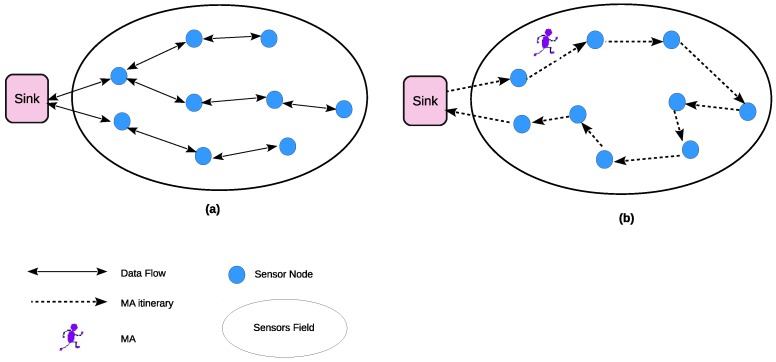
Data gathering based on: (**a**) Client–Server. (**b**) mobile agent (MA).

To overcome the above issues, recently, mobile agent (MA) has been used as an efficient tool for data collection and aggregation in WSNs [4,5,6,7]. In WSNs, MA can be defined as a computational code that is injected inside a packet [8], and this packet is dispatched from the sink and migrates among the nodes to perform a particular task(s) autonomously (i.e., data aggregation) [9,10]. Figure 1 shows the data gathering process based on MA and client–server architecture. In Figure 1b, the MA roams the network and collects the data from the nodes via single-hop, whereas in Figure 1a, the nodes send their data individually to the sink using single-hop or multi-hop routing techniques.

The use of the MA-based computing paradigm provides several advantages [11,12] in the field of WSNs, including:

1. *Local data processing:* the MA can migrate from node to node and do a local processing at the node in order to achieve an assigned task on behalf of the MA’s dispatcher (sink), and then it returns to the sink with the results. This would lead to a decrease in the network’s bandwidth because the nodes no longer need to transmit their data frequently to the sink for data processing.
2. *Extensibility and task adaptability:* Within the same network, several MAs with different assigned tasks can be dispatched to the network. Each MA can be used to carry out a specific task, and then different applications can be achieved. Therefore, the extensibility and task adaptability of MA extends WSNs’ functionality.
3. *Fault-tolerance:* The itinerary of MA can be dynamically determined by the information gain and energy constraints [6]. The MA can check the information of the next hop nodes before it decides to migrate to the next hop. Here, the fault-tolerance such as link failure or dead nodes can be avoided during the MA path and then protect the MA from being lost.
4. *Progressive accuracy:* During the migration, the MA carries a partially integrated result obtained from nodes that have already been visited by the MA. Thus, assuming the MA follows an itinerary determined based on the information gain, the migration from node to node is constantly increasing the accuracy of the integrated result. Therefore, the MA can terminate its migration and returns the results when the accuracy of the integrated result satisfies a given threshold. This advantage of MA decreases both network bandwidth and computation time by avoiding visiting unnecessary nodes.
5. *Reliability:* The MA can be dispatched when the network connection is active and it returns the results when the network connection is re-established. Consequently, the MA’s performance is not affected much by the reliability of the network.

In WSNs based on MA, the MA design can be broken down into four components [13]:

1. Architecture
2. Itinerary planning
3. Middleware system design
4. Agent cooperation

Among the MA design components, the itinerary planning has a direct impact on the energy consumption. The itinerary planning of the MA is the order determination of the source nodes to be visited during the MA migration. Finding the optimal itinerary of the MA has been identified as an NP-hard problem [6]. Along this line, itinerary planning algorithms have been developed to determine sub-optimal MA itinerary. The itinerary planning algorithms can be classified into single itinerary planning (SIP) and multi itinerary planning (MIP) [14]. In SIP, only a single MA is dispatched from the sink to visit the source nodes. The SIP algorithms [4,5,6,15] proposed earlier only performed well in small- or medium-scale sensor networks. However, in a large-scale networks, SIP incurs the following drawbacks [14]:

- Long delays because of migrating to hundreds of source nodes.
- An increase in the MA’s packet size due to the aggregation of data from a huge number of visited source nodes.
- Low reliability when the MA accumulates a huge amount of data.
- The probability of losing the MA’s packet increases when a single MA visits many source nodes.

In order to overcome the drawbacks of SIP, multi itinerary planning algorithms [16,17,18] were proposed. In MIP, several MAs are distributed to the network. The MAs work concurrently to visit groups (partitions) of source nodes. Each MA is assigned to one partition within a shorter itinerary, where it migrates to a subset of source nodes (within a partition) to perform the data aggregation task. The dispatched MA carries its processing code (e.g., aggregation code) from the sink to the assigned partition to collect the data from the source nodes. On completing its task, it returns to the sink with the accumulated data. The size of the accumulated data by the MA varies from one partition to another, which depends on the number of source nodes within each partition. This process enables a reduction in the MA packet size, which further leads to a decrease in the energy consumed as compared to the SIP algorithms. Moreover, due to the distribution of aggregation tasks among multi MAs, the task duration is minimized (lower delay). Although the MIP did overcome the weaknesses of the SIP algorithms, its design is complicated due to the introduction of several challenging issues [11], which include:

- Determining the optimal number of MAs.
- Partitioning the source nodes into subsets of groups and assigning each MA to a specific group.
- Finding the optimal itinerary of each MA.

A greatest information in the greater memory-based MIP (GIGM-MIP) approach was proposed in [19]. In the GIGM-MIP approach, each partition may have more than one MA. The data size of the source nodes in each partition will determine how many MAs will be dispatched to that partition. Although this solution has balanced the accumulated data among MAs, employing more than one MA in one partition results in an increase in the number of MA migration hops due to the increased number of itineraries in each partition. Moreover, all the MAs employed in a particular partition have to carry the aggregation code (which is identical for all the MAs) to the target source nodes for the aggregation task. Multiple MAs carrying the same aggregation code within a single partition would result in an increase in energy consumption.

In this paper, a spawn multi-mobile agent itinerary planning (SMIP) is proposed to reduce the substantial increase in cost of energy as well as time used in the data gathering processes. The proposed approach is based on the agent spawning such that the main MA is able to spawn other MAs within a single partition. The spawning MA has different tasks assigned from the main MA, such that it only returns the accumulated data to the sink. The main goal of the proposed SMIP approach is to alleviate the problem of workload due to multiple MAs carrying the same aggregation code within a single partition.

The rest of the paper is organized as follows: Section 2 reviews the related work in this research. Section 3 presents our proposed SMIP approach. Simulation setup is performed in Section 4. Section 5 evaluates the performance of the proposed approach and discusses the simulation results. Finally, Section 6 concludes this paper with future research directions.

## 2. Related Work

This section presents a review of some of the existing MIP algorithms. It is noted that MIP algorithms can be classified into homogeneous networks with one sink [16,17,18,19,20,21], and heterogeneous networks with multiple sinks [22] (based on network topologies). The majority of the existing MIP algorithms are based on the homogeneous network. As such, the works studied in this paper are centered around the homogeneous networks, where the itinerary of each MA is static and predetermined at the sink.

The near-optimal itinerary design (NOID) algorithm [21] was proposed to find the optimal number of MAs in MIP. This algorithm iteratively groups the sensor nodes in the network to separate sub-trees that are connected progressively to the processing element (PE) or sink. After NOID finishes constructing sub-trees, the sink dispatched one MA to each sub-tree. An enhanced version of the NOID algorithm termed the second near-optimal itinerary design (SNOID) algorithm was proposed in [23]. SNOID differs from NOID by considering the nodes’ communication cost when constructing the MA itinerary. The number of MAs in SNOID is determined by partitioning the area around the sink into concentric zones. The nodes lying within the radius of the first zone around the sink will be the starting points of each itinerary. The first zone radius can be obtained by ${ar}_{max}$, where *a* is an input parameter in the range [0, 1] and $r_{max}$ is the maximum transmission range of any sensor node. Each MA itinerary starts from the zones close to the sink and extends to the outer zones. Similarly, a meta-heuristic method called iterated local search (ILS) was further proposed in [24]. This algorithm is like the other tree-based MIP algorithms (NOID and SNOID), but it differs in considering the increase in MA’s packet size as well as the energy consumption due to migration over intermediate nodes when it constructs the MA itinerary. Although tree-based MIP algorithms perform better than SIP algorithms, the itinerary of the MA consumes extra energy due to the reverse routes that the MA take—especially when there are a huge amount of branches.

The central location-based MIP (CL-MIP) algorithm was proposed by [16], where the determination of the optimal number of MAs in MIP can be divided into four parts; (1) Visiting central location (VCL) selection algorithm, (2) Source grouping algorithm, (3) Determining the source-visiting order using SIP algorithms, (4) Iterative algorithm to ensure that all source nodes have been assigned to their MAs. The main idea of VCL algorithm is to calculate the source nodes density by the distribution of an impact factor among the source nodes. So, if *n* represents the number of source nodes, then each source node will receive (n−1) impact factor from others, and one from itself. Then, the location of the source node with the largest accumulated impact factor will be selected as a VCL (the density of the source nodes). After selecting the VCL, all the source nodes within the radius of VCL are grouped together and assigned to an MA. By using the iterative algorithm, the above process is repeated until all the remaining source nodes are grouped and assigned to the MAs. Finally, each itinerary is determined by using one of the SIP algorithms. Nevertheless, CL-MIP algorithm assumes a cluster-based approach, where the source nodes are arranged geographically and distributed in several clusters. This limits the use of the algorithm when the sensor nodes are sparsely deployed. Moreover, CL-MIP algorithm can result in source nodes isolation (few source nodes are located together) and then assigned a new itinerary. This would lead to an increase in energy consumption, especially when the isolated source nodes are located far from the sink.

Motivated by the need to determine the optimal number of MAs in MIP, a directional source grouping-based MIP (DSG-MIP) algorithm was proposed in [20]. This algorithm partitions the network area into sector zones, whose centers consist of a sensor node that lies within the sink’s radius zone. The sink zone can be obtained by the method presented in the SNOID algorithm in [23] . The contribution in the DSG-MIP algorithm is to establish an angle gap to partition the network into sectors. The angle gap is determined by the nodes density described in [16]. A particular angle gap threshold is used to determine the sector’s size. After several iterations, the isolated source nodes may be arranged to a new sector (with new itinerary). Here, instead of adding these isolated source nodes to a new sector, it can be inserted into existing sectors by expanding the angle with the consideration of the metric of shortest distance to existing itineraries. However, this solution increases the delay of the MA when the isolated source nodes are located far from the existing itineraries. Additionally, determining the optimal gap threshold in the DSG-MIP algorithm is still regarded as a challenging issue.

Similarly, a genetic algorithm (GA)-based MIP was proposed in [17] to also determine the optimal number of MAs in MIP. The aim was to deal with MIP as a single problem instead of utilizing the four-parts in the outlined in CL-MIP [16] and DSG-MIP [20] algorithms. In GA-MIP, a GA is used to determine the number of MAs and their assigned source nodes by two-level coding method. The coding represents a gene that contains source-ordering-code (sequence array) and source grouping code (group array). A source-ordering-code is an array that includes segments, where each segment has a number of source nodes to be visited by a particular MA. A source grouping code is an array of numbers, with each number specifying the number of source nodes of each segment in the source-ordering-code. The two basic operations of GA (crossover and mutation) are used in each iteration, and a fitness function is adapted to select the better genes to survive. GA-MIP has better performance than other previous MIP algorithms in terms of delay and energy consumption, but it is a greedy approach which produces a substantially suboptimal MIP solution and high computation complexity.

In most of the previously proposed MIP algorithms, the geographic information of the sensor nodes is the main parameter used to determine the optimal number of MAs and their itineraries. Recently, a greatest information in the greater memory-based MIP (GIGM-MIP) algorithm was proposed in [19]. This algorithm not only considers the geographic information, but also takes into account the data size in each partition to formulate the optimal number of MAs and their itineraries. In GIGM-MIP, k-means algorithm is used to partition the network into K clusters (partitions). After partitioning the network, GIGM-MIP calculates the data size of the source nodes in each partition. This data size will then determine how many MAs would be assigned to that partition such that each partition may have more than one MA. However, GIGM-MIP algorithm has two main drawbacks. The first one is that with k-means partitioning algorithm, the number of the partitions has to be manually identified by the user. This means that the partitioning of the network is not accurately obtained. The second limitation of the GIGM-MIP algorithm is that the distribution of more than one MA to a single partition would increase the number of MA’s hops. Moreover, if the partition has more than one MA, each MA has to carry the processing code (data aggregation code) to its target source nodes. This limitation has caused an increase in energy and time consumption.

## 3. Spawn Multi-Mobile Agent Itinerary Planning (SMIP) Approach

### 3.1. Partitioning the Network

In the GIGM-MIP algorithm, the k-means algorithm was used to partition the network. As mentioned earlier in Section 2, the algorithm inaccurately partitions the network. As such, an x-means algorithm [25] was adopted in this paper for clustering. The x-means algorithm is an extension of k-means algorithm. The main idea of x-means algorithm is to automatically determine the number of clusters based on Bayesian information criterion (BIC) scores, which is a known selection criterion model. X-means starts with one cluster at the first iteration, then after each iteration, x-means goes into action to make local decisions about which subset of the current centroids should be split in order to better fit the data. The splitting decision is achieved by computing the BIC scores. It is important to remark that the implemented partitioning algorithm in this paper is not the main focus, but rather the itinerary planning of the MAs.

### 3.2. Spawn Mobile Agent (SMA) Algorithm in SMIP

Once the partitioning of the network is done, the sink node determine the number of the MAs, as well as their itineraries for each partition. In the previous MIP approaches, each MA starts its migration from the sink and returns back to the sink with the accumulated data after completing the gathering process. This means that the sink is the start and end point of each MA itinerary. In this article, a spawn multi-mobile agent itinerary planning (SMIP) approach is proposed. The spawn mobile agent (SMA) algorithm is based on one of the characteristics of the agent, named *agent spawning* [26], employed in the proposed SMIP. Agent spawning is the ability to create a new agent that has different capacities and capabilities that are contrary to the original agent. The spawning agent’s task is to handle a part of the tasks at hand of the original agent. Therefore, in this work, we adopt this characteristic (agent spawning) in order to distribute the data gathering task among the MAs such that some MAs have different assigned tasks from others. The sink node assigns one main MA to each partition. The main task of the main MA is to collect the data from the source nodes, and it also has the ability to spawn the new MA (SMA) to do a different task at a certain point. Here, the task of the SMA is only to carry the accumulated data of the main MA back to the sink. As a result of this spawning action, two types of itineraries are defined: main MA itinerary and SMA itinerary.

**Figure 2:**
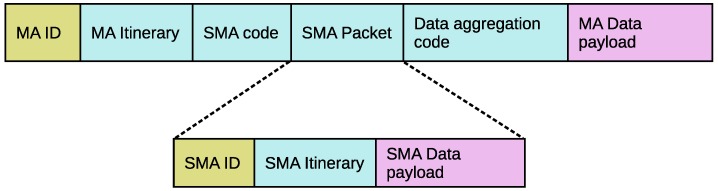
Main MA packet structure. SMA: spawn mobile agent.

It should be noted that the SMA is an entity built inside the main MA packet. The packet structure of the main MA is described in Figure 2. The main MA packet is defined as an entity of six attributes: MA ID, MA itinerary, data payload, SMA code, SMA itinerary, and data aggregation code. The description of these attributes is as follows:

- MA ID: is the identification number of each MA dispatched by the sink node.
- MA Itinerary: contains the itinerary information (source nodes’ visited order list) assigned by the sink node when dispatched.
- Data payload: MA’s data buffer which carries the aggregation data results.
- SMA code: is the code of the spawning carried by MA.
- SMA Packet: includes the SMA ID, the itinerary information of the SMA (visited sensor nodes) to get back to the sink node, and SMA payload data. Note that the MA could carry more than one SMA.
- Data aggregation code: is the implementation of the data aggregation algorithm.

### 3.3. Determining the Itinerary for Each MA in SMIP

In MIP, determining the itinerary for each MA is a challenging issue, where the optimal itinerary has a direct impact on both energy and time consumption. In the proposed SMIP approach, there are two types of MA (main MA and SMA); therefore, the sink is required to determine the itinerary for each MA. At the first, after the network partitioning process is done, the sink assigns one main MA to each partition. In each partition, the source nodes’ visited order list for the main MA is statically determined at the sink by adapting the local closed first (LCF) algorithm. In this paper, LCF algorithm has been adapted for two reasons. First, LCF algorithm has simplicity and low computation process to find the path of MA. Secondly, in terms of fair comparison, this paper adapts the same algorithm that has been used by GIGM-MIP algorithm. LCF algorithm uses the current global network information of all sensor nodes and determines an efficient MA itinerary at the sink before MA is starts its migration. When the main MA is at the sink, LCF lookup to the source node with the shortest distance to the sink from the rest of the source nodes. Then, the LCF algorithm lookup again to the source node with the shortest distance to the current location of the main MA and so on until all the remaining source nodes are assigned to the main MA’s visiting list. After the main MA’s visiting list has completed, the sink then determines in which source node the main MA will create an SMA. In the proposed SMIP approach, the size of the main MA’s data payload is used to determine at which source node the SMA can be created. Specifically, a threshold has been used for the main MA’s data payload, so once the accumulated data exceeds the threshold, the main MA spawns a new SMA. Let S∈(1,2,3,…,i) represent a set of source nodes to be visited by the main MA, then the size of the main MA’s data payload at source node *i* by the MA-assist local reduction process can be calculated as:

(1) $$R_{i}=S_{data}^{i}\cdot \left(1-r\right)$$

where $R_{i}$ is the data reduced at source *i* , $S_{data}^{i}$ is the size of raw data at source *i*, and *r* is the reduction ratio (0 < *r* < 1). When the main MA completes the reduction process at source *i*, it migrates to the next source node (*i* + 1) to perform the same reduction process and then aggregates the result with the one that is already carried from source *i*. Therefore, the size of accumulated data after the MA leaves source *i* can be calculated as follows:

(2) $$\begin{array}{c} S_{ma}^{1}=R_{1},\\ S_{ma}^{2}=R_{1}+\left(1-f\right)\cdot R_{2}\\ S_{ma}^{i}=R_{i}+\left(1-f\right)\cdot R_{i}\\ =R_{1}+\sum_{i=2}^{S}\left(1-f\right)\cdot R_{i} \end{array}$$

where $S_{ma}^{i}$ is the size of the accumulated data after the main MA leaves the source node *i*, *f* is the aggregation ratio (0 ≤ *f* ≤ 1), and $R_{i}$ is the amount of data aggregated by *f*. Note that in Equation (2), there is no data aggregation at the first source node. The itinerary of the SMA is also determined by using LCF algorithm. The only difference between the main MA and SMA itinerary is that the starting point of the SMA is the location where it has been created, while the starting and ending point of the main MA is the sink. Moreover, there is no data aggregation process along with the SMA’s itinerary, because it only visits intermediate nodes. However, LCF algorithm does not always guarantee a lower cost, since the output of this algorithm is highly dependent on the MA’s current location and the size of the accumulated data carried by the main MA. The last source nodes to be visited by the main MA are typically associated with high migration cost due to the increase in the size of the MA’s data payload. Therefore, in the proposed SMIP approach, the data payload is drained and sent back to the sink with the SMA, which would lead to a decrease in the migration cost of the main MA.

It should be noted that in the proposed SMIP approach, the procedure of determining the number of main MAs, SMAs, and their corresponding itineraries utilized for data gathering process is executed centrally at the sink. Thus, since the sink assigns one main MA to each partition, the number of main MAs that will be dispatched to the network is equal to the number of the network’s partitions. While the total number of SMAs in the whole network can be calculated as below:

(3) $$TotalSMAs=\sum_{p=1}^{z}\mathrm{Number}\;\mathrm{of}\;\mathrm{SMAs}\;(p)$$

where *p* is the partition number and *z* is the total number of partitions in the network, NumberofSMAs(p) is the number of SMAs in partition *p*, which is given by:

(4) $$Number\;of\;SMAs\left(p\right)=\frac{TDS_{p}}{MA_{DP}}-1$$

where $TDS_{p}$ is the total data size in partition *p* which can be carried by the main MA using Equation (2) and $MA_{DP}$ is a threshold of the data payload of the main MA. Note that the result in Equation (4) has been reduced by 1 because the last data payload will be carried by the main MA. The pseudo-code of SMIP approach is detailed in Algorithm 1. 

**Algorithm 1:** Pseudo-code of SMIP approach.
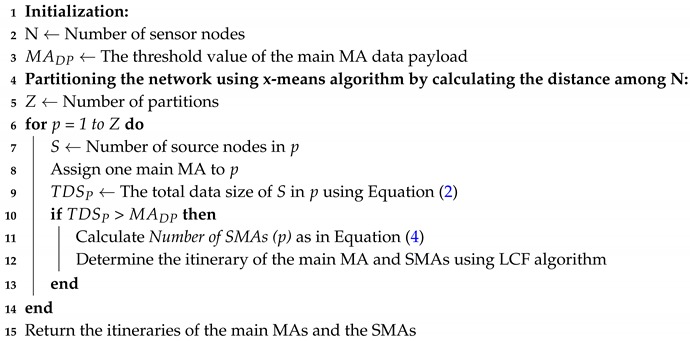


**Figure 3:**
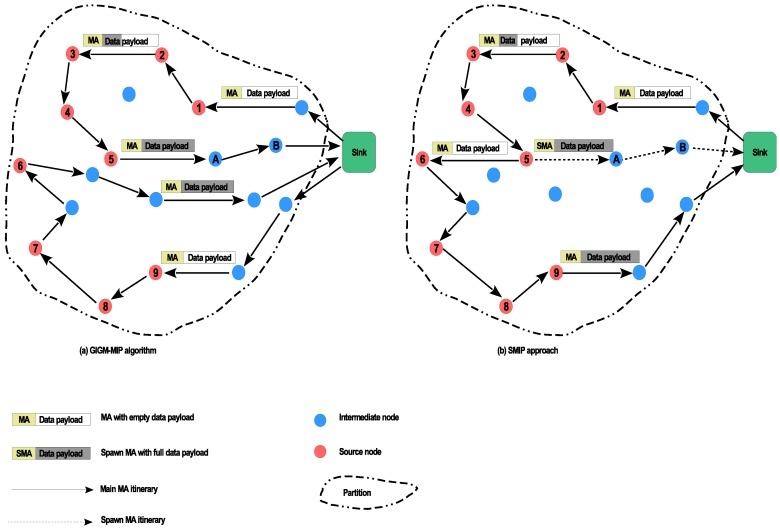
An example of data gathering based on: (**a**) greatest information in the greater memory-based MIP (GIGM-MIP); (**b**) spawn multi-mobile agent itinerary planning (SMIP) approach algorithm.

In order to have a good understanding of the proposed SMIP approach, Figure 3 illustrates an example of data gathering based on SMIP approach when compared to GIGM-MIP algorithm. In this example, the sink is required to send the MA to one partition. In this partition, there are nine source nodes (the red nodes **1**, **2**, **3**, **4**, **5**, **6**, **7**, **8**, and **9**) to be visited by the MA. In Figure 3a, with the GIGM-MIP algorithm, the sink determined that two MAs should be dispatched and visit the nine source nodes. By adapting the LCF algorithm, the source nodes’ visited order of the first MA is (**1**, **2**, **3**, **4**, and **5**). After the first MA complete data collection from source node (**5**), it returns to the sink due to the full size of the data payload. The source nodes’ visited order of the second MA is (**9**, **8**, **7**, and **6**). Note that in the GIGM-MIP algorithm, each MA has to carry its processing code (aggregation code). Moreover, each MA has its own itinerary such that each MA starts its migration from the sink and returns back to the sink with the accumulated data. On the other hand, Figure 3b shows the data collection process of the proposed SMIP approach. With the SMIP approach, the sink dispatches only one main MA to perform the data collection task. The source nodes’ visited order of the main MA as determined by LCF algorithm is (**1**, **2**, **3**, **4**, **5**, **6**, **7**, **8**, and **9**). When the main MA finishes collecting data from source node (**5**) and its data payload exceeds the threshold, the main MA spawns an SMA in order to carry the accumulated data back to the sink. The SMA follows a different itinerary towards nodes **5**, **A** and **B** until it reaches the sink. Note that the nodes along the SMA itinerary work only as intermediate nodes, which means there is no data collection process along the SMA itinerary. Once the main MA spawns the SMA, it continues its migration to perform the data collection task from the remaining source nodes (node numbers **6, 7, 8,** and **9**). Here, if the data payload of the main MA again exceeds the threshold of data payload and there are no more source nodes, the main MA returns the accumulated data back to the sink without making spawn SMA; else, the main MA repeats the above spawning process.

Comparing the GIGM-MIP algorithm against the proposed approach (as shown in Figure 3a), the total hops (including intermediate and source nodes) utilized by the two MAs in the GIGM-MIP algorithm is 20 hops, while in Figure 3b, the number of hops utilized by the main MA and SMA in the proposed SMIP approach is 17 hops. Therefore, the proposed SMIP approach eliminates three unnecessary hops, which has a direct impact on the energy consumption and task duration. Moreover, with the proposed SMIP approach, the MA carries the processing code (which is indispensable) only once to each partition. This has enabled the proposed SMIP approach to save more energy when compared to GIGM-MIP algorithm.

### 3.4. SMA Itinerary Energy Calculation

A WSN is defined as a complete graph G=(V,E) [11], consisting of a set of *N* vertices, |V|=N, where each vertex *i*, (i=0,1,2,…,N−1) in *V* corresponds to a sensor node (SN), $S_{i}$(i=0,1,2,…,N−1), and each edge L(i,j) in *E* corresponds to a wireless communication link between each pair of sensor nodes $SN_{i}$ and $SN_{j}$. The node $S_{0}$ represents the sink node, which dispatches and receives the MAs. Furthermore, there are *M* source nodes included in a specific subset $K^{\prime }$, where $K^{\prime }\in S$.

The wireless communication link L(i,j) is associated with a link cost C(i,j),i,j∈(0,1,2,⋯,N−1)—a function of the power loss to transfer the packet from Si to Sj. Given the cost matrix C=C(i,j)||L(i,j)∈E, in MIP, the MA routing problem is to find a set of *t* near-optimal itineraries, $I=I_{1},I_{2},\ldots ,I_{t}$ so that the overall cost of all the itineraries in *I* is reduced. Each particular *I* will cover a group of sensor nodes $K^{\prime }$ which is a subset of *S*. In order to avoid the interference, each two itineraries should share no common source nodes. The process of the LCF algorithm is to assign the source nodes to an MA’itinerary one by one depending on the shortest distance to the current location of the MA. Then, this process is repeated again for the second MA’itinerary and so on until all the remaining source nodes are assigned to all MAs’ itineraries. In this case, each MA’itinerary will have a different set of source nodes from other MA’itineraries. Each MA’itinerary has a migration energy cost. The total energy cost of all the itineraries can be written as:

(5) $$Ctotal=\sum_{t=1}^{\left|I\right|}IC^{t}$$

where $IC^{t}$ is the energy cost of itinerary $I^{t}$ covered by the MA, and $IC^{t}$ can be simplified to:

(6) $$IC^{t}=\sum_{j=1}^{|I^{t}|}\left(jdf+pc\right)c_{i,j}$$

$|I^{t}|$ represents the number of visited nodes in the itinerary $I^{t}$ by the MA, *j* is the visited sensor node, jdf means the size of data collected by the MA at sensor node *j* after aggregated by a ratio *f*, pc is the MA’s initial size (processing code plus MA packet header), and $c_{i,j}$ is the energy consumption of the MA to migrate from sensor node *i* to sensor node *j*. Note that *j* could act as source node (has data to be collected by the MA) or intermediate node (only hop).

In order to calculate the total energy cost in our proposed SMIP approach, the energy cost of both types of itineraries (main MA itinerary and SMA itinerary) has to be taken into account. Therefore, the total energy cost of SMIP approach can be calculated as follows:

(7) $$C_{total}SMIP=\sum_{t=1}^{\left|I\right|}IC^{t}+\sum_{h=1}^{|I_{SMA}|}I_{SMA}C^{h}$$

where $I_{SMA}C^{h}$ is the energy cost of itinerary $I_{SMA}^{h}$ covered by the SMA. So, $I_{SMA}C^{h}$ can be simplified to:

(8) $$I_{SMA}C^{h}=\sum_{m=1}^{|I^{h}|}\left(m+pc_{SMA}\right)c_{m,j}^{SMA}$$

where $|I^{h}|$ refers to the number of visited nodes in the itinerary $I^{h}$ by the SMA, *m* is the visited sensor node, $pc_{SMA}$ is the SMA’s initial size (processing code plus SMA packet header), and $c_{m,j}^{SMA}$ is the energy consumption of the SMA to migrate from sensor node *m* to sensor node *j*. It should be noted that *m* in Equation (8) acts only as an intermediate node, which means there is no data to be collected along the SMA itinerary.

## 4. Simulation Setup

In order to evaluate our proposed SMIP approach, we have compared the proposed SMIP approach against GIGM-MIP algorithm [19] as well as to the basic MIP algorithm (CL-MIP algorithm). The implementations have been tested using a simulation developed via MATLAB R2014a. The implementations have been tested using simulation developed via MATLAB R2014a. In this paper, MATLAB was chosen for two reasons. First, it is an easy-to-use mathematical simulation tool for different mathematical models and analysis of results. Secondly, in order to fairly compare the simulation results, MATLAB is used because it was used by GIGM-MIP algorithm, which is the compared algorithm to our proposed SMIP approach. We adopted the same network model in [16,17,19,20], which is the most popular network model in data gathering-based MIP. We used the same energy consumption model as in [17,27]. The sensor nodes are static, densely and uniformly deployed within a 1000 m × 500 m network size. We selected a large-scale network with 800 sensor nodes in order to validate the scaling of the proposed SMIP approach. The sink node is located at the center of the network, and it has unlimited energy supply and higher computational capability. All the sensor nodes have the same initial energy with fixed transmission range. Each sensor node has at least one neighbor node with its transmission range set to 60 m. In each data gathering task, a random number of source nodes is selected which is varied from 10 to 40 by the step of 5. Each compared MIP algorithm was tested on the same selected number of source nodes. The sink node has all the geographic information of the sensor nodes, and it is responsible for calculating the main MAs and SMAs itineraries for each data aggregation task. The itineraries are static, where the list of the visited sensor nodes is predetermined at the sink. The simulation parameters are listed in Table 1.

## 5. Performance Evaluation

In this section, we evaluate five performance metrics: task energy consumption, task duration, energy-delay product (EDP), hop count, and task distance. For each data point in each presented figure, we take the average of 30 simulation runs with a different selected random number of source nodes. The proposed SMIP approach was compared against the GIGM-MIP algorithm as well as the CL-MIP algorithm. For each MA itinerary, local closed first (LCF) algorithm was used to determine the visited source nodes order. The LCF algorithm searches for the next node with the shortest distance to the current node. The sink node is the starting point of the main MA itinerary, while the starting point of the SMA itinerary is the point where the main MA made spawn. The sink node is the ending point for both itineraries (main MA and SMA).

**Figure 4:**
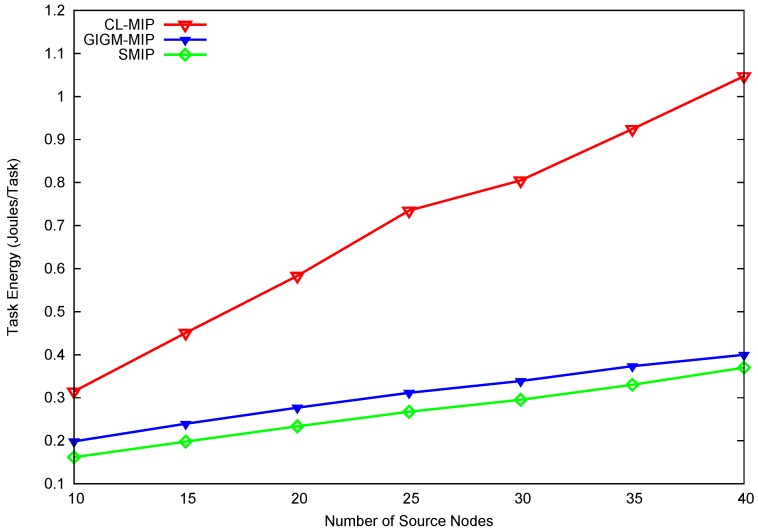
The impact of number of source nodes on energy consumption.

Figure 4 shows the impact of the number of source nodes on the task energy consumption. The task energy consumption is the energy spent for transmitting, receiving, and exchanging control messages to perform the data collection task from all source nodes. In this figure, when the number of source nodes increased, the energy consumption of the CL-MIP algorithm goes high from 0.32 to 1.5 J/Task. The CL-MIP algorithm consumes high energy due to the distribution of a large number of MAs to the network which leads to an increase in the number of MA’s hops. GIGM-MIP algorithm consumes less energy than CL-MIP algorithm when the number of source nodes increases. This is because GIGM-MIP algorithm distributes the data gathering task among the MAs in each partition. On the other hand, the proposed SMIP approach achieves 61.8% and 13.2% energy decrease when compared to CL-MIP and GIGM-MIP, respectively. Significantly, this achievement is obtained for two reasons. The first is the minimization in the number of MA hops. The second reason is that the processing code (aggregation code) of the main MA is carried once to each partition, while in GIGM-MIP algorithm, each partition may have multiple processing codes carried by the MAs. Additionally, in SMIP approach, the accumulated data of the main MA is sent back to sink separately by the SMA, while in the GIGM-MIP algorithm, each MA has to carry its accumulated data to the sink.

In a SIP algorithm, the task duration is the average delay from the time when the MA is dispatched by the sink to the time when MA returns back to the sink. In MIP algorithms, however, since multiple MAs are dispatched to the network and work simultaneously, the task duration of the MIP algorithm is the delay of the MA that returns back to the sink at last. In the proposed SMIP approach, the task duration is the delay of either main MA or SMA that returns back to the sink at last.

**Figure 5:**
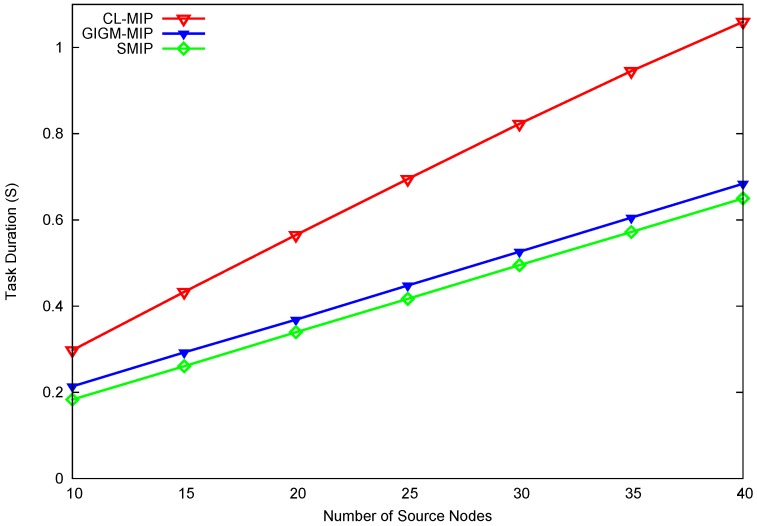
The impact of the number of source nodes on task duration.

As depicted in Figure 5, the proposed SMIP approach achieves a significant improvement in terms of task duration. This improvement is about a 39.4% and 7.04% decrease of task duration as compared to CL-MIP and GIGM-MIP algorithms, respectively. In both CL-MIP and GIGM-MIP algorithms, each MA should travel along its itinerary starting the migration from the sink to perform the data collection process from the source nodes, and then returns back to the sink with the accumulated data. This process increases the number of MA’s hops which leads to a large delay, especially when the source nodes are distributed sparsely over the network. Meanwhile, the proposed SMIP approach reduces the task duration by constructing the shortest MA itineraries with fewer MA hops.

**Figure 6:**
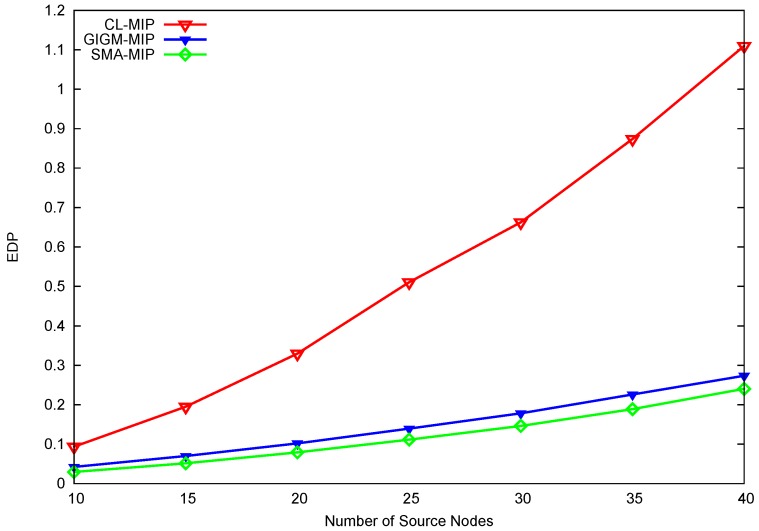
The impact of the number of source nodes on energy-delay product (EDP).

For time-sensitive applications (e.g., wireless multimedia sensor networks and video sensor networks [28]), it is important to consider both energy consumption and task duration (energy-delay product, EDP) in order to evaluate the performance of the proposed approach. EDP can be calculated as EDP = energy × delay, such that the smaller the value of EDP, the better performance obtained. Figure 6 illustrates the overall EDP performance of CL-MIP, GIGM-MIP, and SMIP. Due to the high energy and task duration, CL-MIP has the largest value of EDP, which yields the worst overall performance when compared to GIGM-MIP and SMIP. Evidently, the proposed SMIP approach outperforms GIGM-MIP with a decreased EDP value up to 17.8%, and continues to achieve better performance even when the number of source nodes increases. This verifies the effectiveness of the proposed approach.

**Figure 7:**
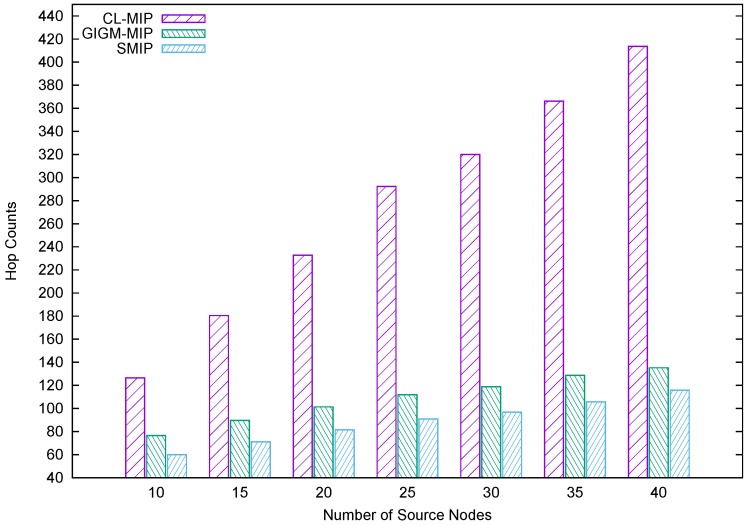
The impact of the number of source nodes on hop counts.

Figure 7 illustrates the accumulated hop counts, which includes both source and intermediate nodes of the compared algorithms. In SIP algorithms, the hop count is defined as the average hop count of the MA itinerary. In MIP algorithms, the average hop count is the accumulated hop counts of all the MAs’ itineraries. As shown in Figure 7, the results indicate that the CL-MIP algorithm produces the largest hop count among other MIP algorithms by constructing many MA itineraries, which returns a large number of hops. Besides, the GIGM-MIP algorithm accumulates fewer hops compared to the CL-MIP algorithm. On the other hand, the proposed SMIP approach has the lowest number of hops due to the shortest distance utilized by the MAs as well as fewer itineraries.

**Figure 8:**
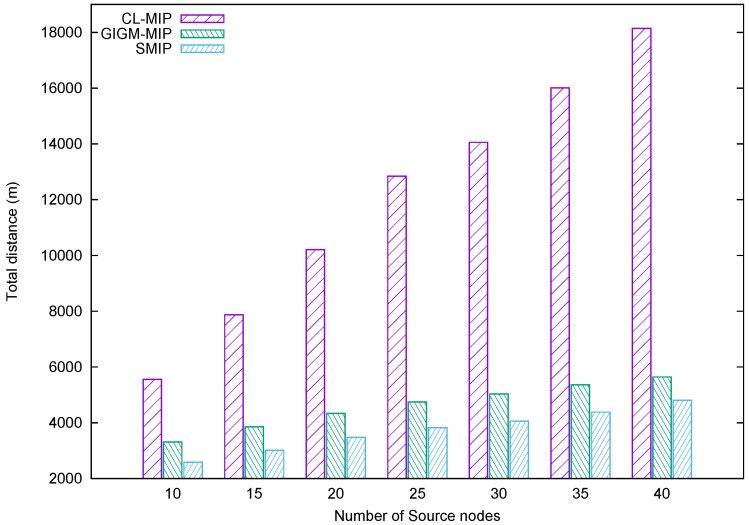
The impact of the number of source nodes on distance traveled by all MAs.

Figure 8 shows the evaluation of the task distance metric. It represents the accumulated distance travelled by all MAs until they return to the sink. When the number of source nodes increases, the accumulated distance spent by all MAs increases due to the construction of many intermediate nodes along with MAs’ itineraries. Note that the intermediate nodes consume more energy than the source nodes due to data forwarding, while the source nodes are visited only once for data collection process. As shown in Figure 8, the proposed SMIP approach achieves the shortest distance compared to GIGM-MIP and CL-MIP algorithms. This achievement is related to the fact that the proposed SMIP approach utilizes fewer intermediate nodes when constructing the MAs itineraries. This indicates that the proposed approach can be suitable for target tracking problems in wireless sensor networks where the distance is a very important factor.

## 6. Conclusions

Determining the optimal number of MAs and their itineraries in MIP has a direct impact on the overall performance of the data gathering task in WSNs. However, the previous MIP algorithms were based on a general assumption such that the sink node is the starting and ending point of each MA’s itinerary. In this paper, a spawn multi-mobile agent itinerary planning (SMIP) approach has been proposed. The main aim of the proposed SMIP was to determine the optimal number of MAs and their itineraries in MIP. The idea of the proposed SMIP approach was based on agent spawning, where the sink node dispatches only one main MA to the assigned partition in the network, and this main MA has the ability to spawn one or more new MAs with different assigned tasks. Furthermore, the proposed SMIP approach used x-means clustering algorithm to partition the network such that the number of clusters is efficiently achieved. Subsequently, extensive simulations have been carried out to evaluate the performance of the proposed SMIP. The results shown that SMIP has achieved significant improvements in terms of energy and task duration. Additionally, the proposed SMIP approach has achieved a better performance (EDP) compared to the previous MIP algorithms. Future research needs to consider fault tolerance (such as sensor’s failure and battery depletion) during the determination of the MA itinerary, so that the MA can dynamically migrate to the next hop node.

## Figures and Tables

**Table 1:** Simulation parameters of SMIP approach.

**Network Parameters**	**Value**
Network’s Terrain	1000 m × 500 m
Number of deployed nodes	800
Number of source nodes	10–40
Transmission range	60 m
Raw data size	1024 bits
**MA Parameters**	**Value**
MA processing code	1024 bits
MA accessing delay	10 ms
Raw data reduction ratio	0.8
Aggregation ratio	0.9
Data processing rate	50 Mbps
Data payload threshold	1500 bits
**SMA Parameters**	**Value**
SMA processing code	128 bits
SMA accessing delay	10 ms
Data processing rate	50 Mbps
